# Immature and Maturation-Resistant Human Dendritic Cells Generated from Bone Marrow Require Two Stimulations to Induce T Cell Anergy *In Vitro*

**DOI:** 10.1371/journal.pone.0006645

**Published:** 2009-08-14

**Authors:** Thomas G. Berger, Hendrik Schulze-Koops, Michaela Schäfer, Ester Müller, Manfred B. Lutz

**Affiliations:** 1 Department of Dermatology, University Hospital Erlangen, Erlangen, Germany; 2 Department of Internal Medicine III, University Hospital Erlangen, Erlangen, Germany; 3 Institute for Virology and Immunobiology, University of Wuerzburg, Wuerzburg, Germany; 4 Division of Dermatology, Tawam Hospital in affiliation with Johns Hopkins Medicine, Al Ain, United Arab Emirates; 5 Division of Rheumatology, Medizinische Poliklinik, Ludwig-Maximilians-University, Munich, Germany; New York University School of Medicine, United States of America

## Abstract

Immature dendritic cells (DC) represent potential clinical tools for tolerogenic cellular immunotherapy in both transplantation and autoimmunity. A major drawback *in vivo* is their potential to mature during infections or inflammation, which would convert their tolerogenicity into immunogenicity. The generation of immature DC from human bone marrow (BM) by low doses of GM-CSF (lowGM) in the absence of IL-4 under GMP conditions create DC resistant to maturation, detected by surface marker expression and primary stimulation by allogeneic T cells. This resistence could not be observed for BM-derived DC generated with high doses of GM-CSF plus IL-4 (highGM/4), although both DC types induced primary allogeneic T cell anergy *in vitro*. The estabishment of the anergic state requires two subsequent stimulations by immature DC. Anergy induction was more profound with lowGM-DC due to their maturation resistance. Together, we show the generation of immature, maturation-resistant lowGM-DC for potential clinical use in transplant rejection and propose a two-step-model of T cell anergy induction by immature DC.

## Introduction

Establishment of immunological tolerance is the ultimate goal for causative treatments of autoimmune diseases, allergies and transplant rejections. While T cells are major effectors in autoimmune responses, DC are not only potent inducers T cell immunity but also control T cell tolerance [1], [2]. There is considerable interest in the generation tolerogenic DC to control autoreactive T cells in human patients [3]. With the diversity of T cell tolerance mechanisms, there might not be a single tolerogenic DC type able to control all of these mechanisms. Therefore it is important to select or generate the optimal tolerogenic DC type that is best suited to tolerize a specific disease.

The various mechanisms of T cell tolerance can be subdivided into T cell-intrinsic mechanisms, such as T cell anergy, deletion, immune deviation and T cell extrinsic control by induction of regulatory T cells (T regs) [4], [5], [6], [7]. T cell anergy induction has been originally described with T cell clones, i.e. cells that have undergone at least one stimulation before employed in the anergy experiments [8]. Subsequent exposure of such primed, but resting CD4^+^ T cells cross-linked to CD3 resulted in an anergic state of these cells. Characterized by their inability to respond to subsequent TCR signals, but ability to maintain proliferative capacity by polyclonal stimulation. Previous studies indicate T cell anergy could be induced by a single application of orally applied ovalbumin antigen [9] or superantigen [10].

Although detailed molecular mechanisms of signal transduction to induce T cell anergy have been identified [4], the physiological type of antigen-presenting cell (APC) inducing anergy has not been investigated. Although it has been postulated that a single hit may not be sufficient to induce T cell anergy the need for a single or sequential trigger of naïve T cells by anergy-inducing APC remains largely unclear [11].

The ability of T cell anergy induction could be achieved with CD3 antibodies in the absence of CD28 costimulation. A cellular counterpart that provides TCR/CD3 signals without CD28 engagement is represented by immature DC, characterized by a low expression of surface MHC II molecules and low to absent levels of costimulation by CD80/CD86. Technically, the preservation of an immature state remains difficult for *in vitro* generated DC since murine or human immature DC spontaneously mature [12]. These cells are highly sensitive to culture conditions [13], or inflammatory, microbial and T cell-derived stimuli [14], [15]. For *in vivo* application of immature tolerogenic DC it is mandatory to block their maturation [16]. A specific maturation block to preserve the immature DC phenotype was first described by treatment of human Langerhans cells with UV light [17]. Thereafter, many other treatments have been reported to inhibit DC maturation, including IL-10, TGF-β, glucocorticoids and vitamin D3 analogues [reviewed in [7]. However, only few reports exist, where immature DC could be generated *in vitro* that were resistant to further maturation signals. These methods include the combined treatment of DC with IL-10 plus TGF-β [18], dexamethasone or rapamycin [19] or vitamin D3 analogues [20]. DC blocked with combined IL-10 plus vitamin D3 treatment have already been applied sucessfully in macaques [21].

Immature DC have been also applied *in vivo* to delay allogeneic transplantations in mice. Despite the positive effect on the graft survival, the delay of rejection was only moderate as DC maturation occurred *in vivo*, shown by B7-2 upregulation [22]. This further underlined the prerequisite of maturation-resistance. In the same allogeneic heart transplantation model, pretreatment of mice with immature and maturation-resistant DC allowed a prolonged graft survival as compared to normal immature DC [22]. These immature and maturation-resistant DC were generated from murine BM cells by using very low doses of GM-CSF (5–20 U/ml) in the absence of IL-4 [23].

Here we show that human bone marrow cells, when cultured with low doses of GM-CSF, give rise to immature DC that do not respond to maturation stimuli anymore, similarly demonstrated in the mouse model. These human immature and maturation-resistant DC should be safer as compared to normal immature DC for tolerance induction in clinical applications of transplant rejection or T cell-mediated autoimmune diseases.

## Results

### Human BM-DC generated under lowGM conditions are maturation resistant

The standard source for the generation of human DC are peripheral blood monocytes, while in the murine system, DC are mostly generated from BM. Previously, we described a method how murine DC can be generated with low doses of GM-CSF from BM cells without undergoing spontaneous maturation. These lowGM-DC remained at their immature state and were resistant to stimulation with TNF, LPS or CD40 ligation [23]. As maturation-resistance is advantageous for the clinical application of tolerogenic DC, we investigated whether such DC could be generated from human BM.

**Figure 1:**
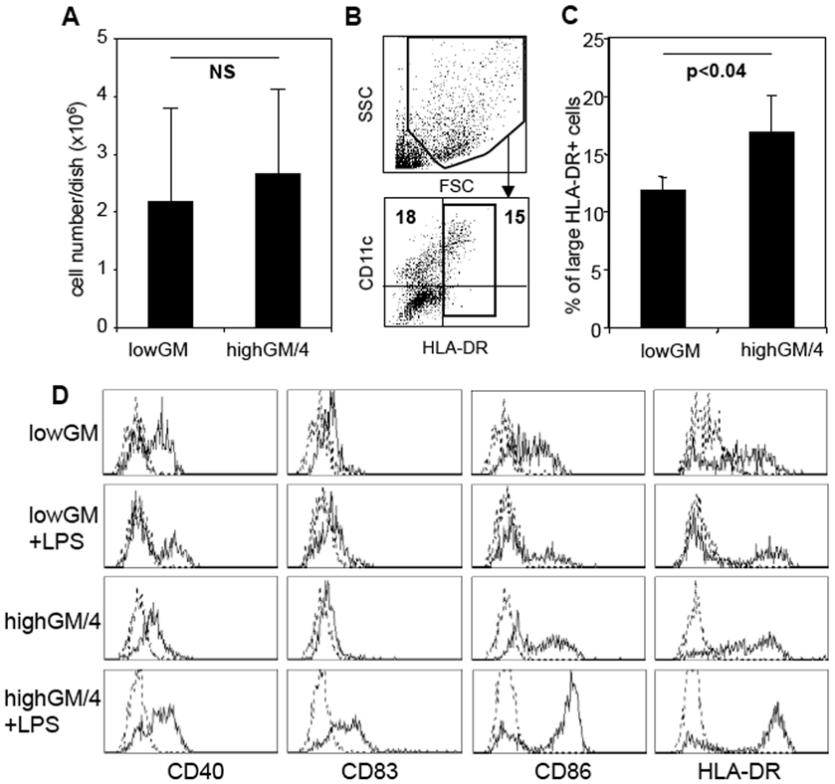
Human BM-derived lowGM-DC do not upregulate surface markers upon maturation. Human BM cells were cultured under standard conditions with 800 U/ml GM-CSF plus 250 U/ml IL-4 or under lowGM conditions with 5 U/ml GM-CSF for 6 days in RPMI+1% AB plasma. A. The cells were harvested and counted under trypan blue exclusion. The mean values±standard deviations of 8 independent experiments are shown. B. The harvested cells were analysed by flow cytometry by setting gates on large cells in the FSC/SSC profile and HLA-DR^+^ cells. C. Analysis of 3 independent experiments as gated in B. Mean values±standard deviations are shown. D. The cells were then exposed to 100 µg/ml LPS for 24 h to induce maturation or left untreated and surface stained for the indicated markers (straight lines) or isotype controls (dotted lines). Cells were gated as in B to exclude lymphocytes and precursor cells. The data from one experiment shown are representative of 5 independent experiments.

Human BM cells were cultured following the murine protocol to generate lowGM-DC. After 6 days, the cells were analyzed for their expression of typical markers for mature DC. LowGM-DC and control DC generated with high doses of GM-CSF plus IL-4 (highGM/4), similar to protocols for human monocyte-derived DC, yielded the same cell numbers after 6 days (Fig. 1A). However, the cellular yields from highGM/4 cultures were slightly higher as compared to lowGM conditions. These cells also expressed low levels of HLA-DR being consistent with an immature DC phenotype (Fig. 1B and C). Together, under either of the two conditions 5×10^6^ BM cells yielded 2.5–4.4×10^5^ DC, respectively. Upon stimulation with LPS only the highGM/4 upregulated CD40, CD83, CD86 and HLA-DR, while the lowGM cells retained their immature DC profile (Fig. 1C). Similar results were obtained by stimulation with a standard maturation cocktail (TNF, IL-1β, IL-6, PGE_2_), CD40 ligation or with the TLR3 ligand Poly I∶C (not shown).

### Matured lowGM-DC remain functionally immature in stimulating an allogeneic MLR

**Figure 2:**
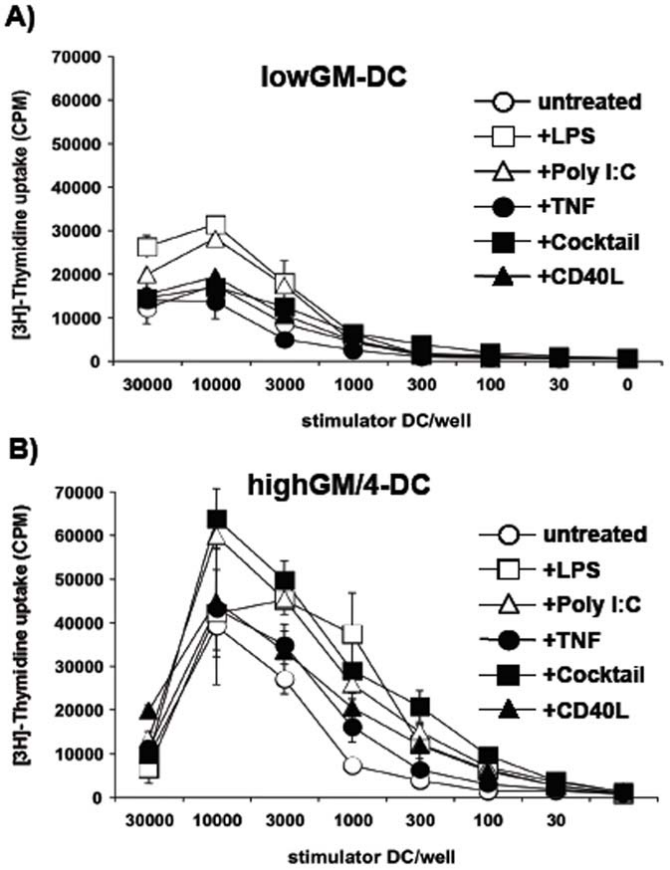
LowGM-DC remain functionally immature after maturation. DC were generated under low-GM (A) and standard highGM plus IL-4 conditions (B) for 6 days, stimulated with the indicated reagents or a “cocktail” consisting of TNF, IL-1β, IL-6 and PGE_2_ for 24 h and then added at titrated numbers to allogeneic T cells, as represented by non-adherent fraction of PBMC of an allogeneic donor. After 3 days [^3^H]-thymidine was added overnight to measure proliferation. The data from one experiment shown are representative of 3 independent experiments. Values represent the mean±standard deviation error bars of triplicate cultures from one experiment.

To further substantiate the phenotypical results, we compared lowGM-DC with highGM/4-DC in their capacity to prime naïve allogeneic T cells. Both types of DC were used as immature cells or after treatment with various maturation stimuli. While immature and mature lowGM-DC showed weak T cell stimulation capacity with the maximal response being less 35,000 cpm (Fig. 2A). In contrast, highGM/4-DC showed a maximal T cell stimulation capacity of 40,000 cpm already by immature DC and could be further upregulated after maturation (Fig. 2B).

### A single stimulation by immature DC does not induce T cell anergy

**Figure 3:**
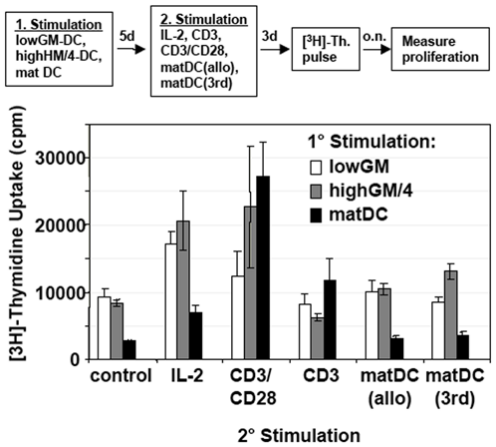
A single allogeneic T cell priming by lowGM-DC does not induce T cell anergy. Allogeneic T cells were stimulated with either immature lowGM-DC or immature highGM+IL-4-DC or LPS-matured highGM+IL-4-DC. After 5 days the T cells were restimulated with IL-2 or the indicated antibodies or LPS-matured DC from the original allotype (matDC allo) or a third party allotype (matDC 3rd) or cells were left without stimulation (control) for another 3 days before [^3^H]-thymidine ([^3^H]-Th.) was added overnight to measure proliferation as shown in the flow chart. The data from one experiment shown are representative of 5 independent experiments. Values represent the mean±standard deviation error bars of triplicate cultures from one experiment.

In our murine experiments, we observed the induction of T cell anergy by a single injection of lowGM-DC into allogeneic mice ([23] and unpublished observations). To tested whether lowGM-DC would be able to induce allogeneic T cell anergy *in vitro*, immature lowGM-DC and highGM/4-DC were generated from BM and compared to LPS-matured DC generated from PBMC. All these three DC types were co-cultured with allogeneic T cells for 5 days. Then the T cells were restimulated with different stimuli, such as high doses of IL-2 or anti-CD3, either alone or in combination with anti-CD28. These conditions were also repeated with mature monocyte-derived DC (Mo-DC) from the same donor as in the first stimulation, or mature Mo-DC from a third party donor. Under all conditions a mild proliferation of T cells could be observed, which was at the same level or slightly higher than the unstimulated T cells (Fig. 3). Thus, T cell anergy was not induced under all of these conditions. Shortening (3 days) or extending (7, 10 days) the time interval before restimulation did not modify this result (not shown).

### Two stimulations by lowGM-DC are required to induce T cell anergy

**Figure 4:**
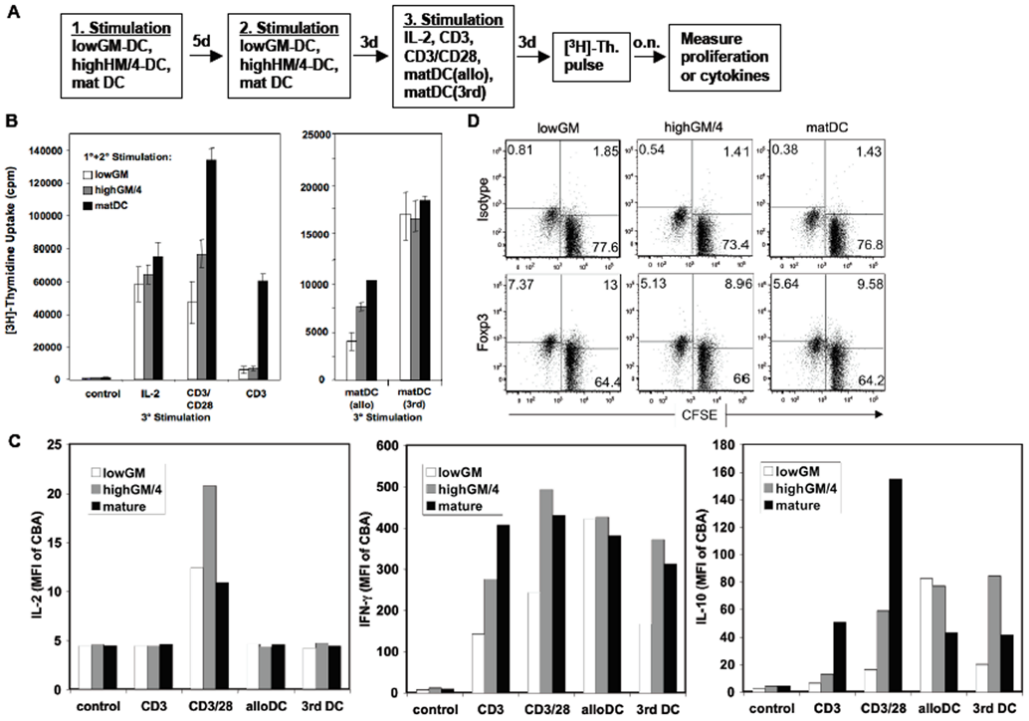
Allogeneic T cell anergy induction requires two stimulations by lowGM-DC. A. Allogeneic T cells were stimulated twice (for 5 days and then for another 3 days) with either immature lowGM-DC or immature highGM+IL-4-DC or mature highGM+IL-4-DC and then restimulated a third time for another 3 days with IL-2 or the indicated antibodies or LPS-matured DC from the original allotype (matDC allo) or a third party allotype (matDC 3rd) or cells were left without stimulation (control) for another 3 days. B. Then [^3^H]-thymidine ([^3^H]-Th.) was added overnight to measure proliferation. The data are representative of 4 independent experiments. C. Supernatants from the cultures shown in Figure 4B were tested for their content of IL-2, IFN-γ, or IL-10 by cytokine bead array (CBA). The relative mean fluorescence values of the FACS analysis are shown. The data from one experiment shown are representative for 3 independent experiments. Values in bar graphs represent the mean±standard deviation error bars of triplicate cultures from one experiment. D. CFSE-labeled T cells were restimulated by the indicated DC type for 4 days and then stained for intracellular expression of Foxp3. Cells were measured by flow cytometry. Gating was performed on CFSE^+^ cells and is plotted against Foxp3 or isotype. CFSE^low^ cells represent allo-responsive proliferated cells.

T cell anergy induction has been proposed by some authors to require a single TCR stimulation in vitro or a single injection of an antigen. However, data by other groups indicated that prestimulation of the T cells would be required to establish T cell anergy [11]. Many T cell anergy experiments described in the literature were performed with antigen-experienced T cell clones. For T cell anergy induction *in vivo*, soluble antigens were injected intravenously. All of these *in vitro* studies employed antibodies for T cell treatment and did not investigate the APC type *in vivo*. The precise type of prestimulation was never assessed. The injected antigens may be endocytosed by immature splenic DC, which present the peptides to T cells and establish their anergic state after about 3 days. It is not clear whether a single T-DC contact is required to result in T cells anergy or several rounds of contacts are needed within this time period. In fact, a single encounter of naïve T cells with immature DC was not sufficient to render them anergic (Fig. 3). To test whether T cell anergy induction in naïve T cells may depend on two stimulations, we cultured the allogeneic T cells twice with the same type of DC. Now T cell anergy induction could be clearly observed with both types of immature lowGM-DC and highGM/4-DC, as indicated by their inability to respond to a TCR signal alone (anti-CD3), by their lowered capacity to respond to full T cell stimulation (allogeneic mature DC and the anti-CD3/antiCD28 combination). Nevertheless their potential to proliferate on polyclonal stimulation by IL-2 or to third party-derived mature Mo-DC was not affected (Fig. 4B). Two repetitive stimulations of the T cells with mature DC resulted in the expected memory T cell phenotype with reduced costimulation requirement, as reflected by the capacity to respond to anti-CD3 alone (Fig. 4B). In addition, T cells stimulated with mature DC and followed by CD3 antibodies only showed a cytokine profile of differentiated cells, such as high IFN-γ, some IL-10 (Fig. 4C), but almost no IL-2. IL-4 was never detected under all conditions (not shown). Importantly, T cells in culture with lowGM-DC and restimulated with anti-CD3 did not produce IL-2 or IL-10 in relevant amounts, only little IFN-γ (Fig. 4C). Also, it did not show higher Foxp3^+^ cell frequencies in proliferated cells (allo-specific) as compared to non-prolifereted cells (not allo-responsive). This was further comparable in cultures stimultated by highGM/4 or mature DC (Fig. 4D). Thus, our data may indicate non-allo-specific Foxp3^+^ regulatory T cells or IL-10 producing T regulatory type-1 cells [24], [25] were induced after two rounds of stimulation with immature lowGM-DC or highGM/4-DC, but argue for the induction of allogeneic T cell anergy.

### The two stimulations by immature DC cannot be substituted by mature DC at any time of stimulation

**Figure 5:**
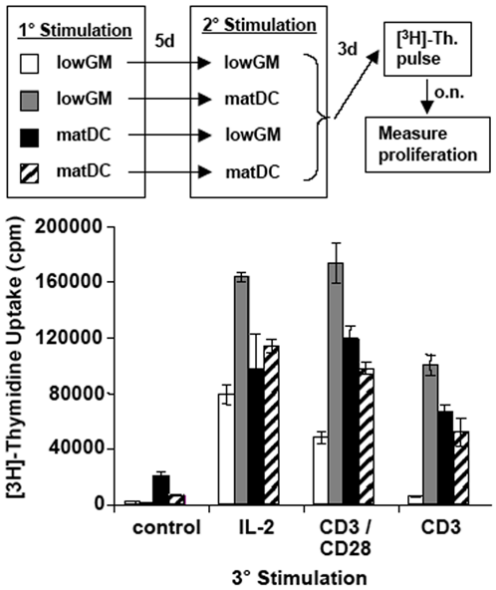
Mature DC cannot substitute for immature DC at either stimulation time points. Immature lowGM-DC or mature highGM/4-DC were generated to stimulate allogeneic naïve T cells. After 5 days the T cells were restimulated with the same type of DC or in an opposite setting as indicated in the legend. After another 3 days the T cells were restimulated with the indicated reagents for 3 days before [^3^H]-thymidine incorporation was detected to measure proliferation. The data from one experiment shown are representative of 3 independent experiments. Values represent the mean±standard deviation error bars of triplicate cultures from one experiment.

Anergy induction in naïve T cells required two stimulations with immature DC, while previous studies required only one stimulation. However they used T cell clones or preactivated T cells. The differential types of prestimulation by antibodies often employed CD3 stimulation without CD28 costimulation. On the other hand the state of activation or preactivation of T cell clones for anergy induction was unclear. Therefore we wondered whether one of the two stimulation could be substituted by mature DC. We exchanged the immature lowGM-DC by mature highGM/4-DC during the first or second round of stimulation. The results indicate that only two subsequent stimulations by immature lowGM-DC induced an absolute unresponsiveness to anti-CD3 and a relative unresponsiveness to anti-CD3/anti-CD28 with no impairment to respond to IL-2 (Fig. 5). These data indicate that both rounds of stimulation require immature DC and cannot be substituted by mature DC at any time point.

## Discussion

Here we described a method to generate human immature and maturation-resistant DC from BM precursor cells which was adopted from a murine protocol [23]. All reagents and media that were used in this protocol were approved for GMP guidelines and thus suitable for human clinical use. LowGM-DC were resistant to common maturation stimuli and induced T cell anergy in naïve allogeneic T cells when stimulated twice with immature DC. HighGM/4-DC were not maturation-resistant and induced T cell anergy, but less profound compared to lowGM-DC. Untreated immature DC are extremely sensitive to all kinds of maturation stimuli, even strong pipetting [26]. The achievement of maturation-resistance is one prerequisite for the clinical application of tolerogenic DC. Plans to introduce tolerogenic DC in human clinical studies involve such maturation-resistant DC [19].

The phenomenon of maturation-resistance have been observed also after treatment of DC with TGF-β plus IL-10 [27], dexamethasone [28], [29] or vitamin D3 analogues [20]. These factors were found to induce a similar maturation-resistant phenotype of DC. The resulting immature DC did either not respond to conventional maturation stimuli or underwent an alternative activation, that results in the production of tolerogenic cytokines (IL-10, TGF-β) or the expression of tolerogenic surface markers (ILT-3) (reviewed in [7]). The maturation stimuli tested on these DC maturation-resistance included TLR ligands and endogenous factors, such as proinflamatory cytokines (TNF) or CD40 ligands, which reflect very different pathways of DC activation. However, the underlying molecular mechanisms of DC maturation-resistance are not fully understood. DC treated with vitamin D3 analogues showed an impaired NF-κB activation leading to IL-12p40 gene repression [30]. When dexamethasone treated DC were stimulated by LPS they showed a reduced IL-12 production but the IL-10 production was not affected [31], indicating that an immunogenic DC maturation is blocked but an suppressive activation pathway remains functional. The effect of vitamin D3 analogues on DC function seems to result in a modified DC phenotype expressing ILT-3, IL-10, and inducing T regs rather than inducing T cell anergy [20]. Whether ILT-3 expression on DC could represent a molecule to distinguish between the induction of CD4^+^ T cell anergy or CD4^+^ T regs remains to be determined. Analyses with CD4^+^ and CD8^+^ T cells strongly argue for a role of ILT-3 on DC in the generation of suppressive T cells [32].

We achieved a similar phenotype of maturation-resistant DC when cord blood CD34^+^ cells were cultured under lowGM conditions, however at lower cell yields (not shown). In contrast, we were unable to apply the lowGM protocol to adherent peripheral blood monocytes as a source of DC precursors (not shown). This may indicate that earlier developmental stages and precursors different from monocytes are required to induce this type of differentiation.

In the murine system, the presence of IL-4 during the generation of lowGM-DC abrogated the maturation-resistant phenotype [23] and during normal highGM cultures can influence the subset generation [33]. When IL-4 and IL-13 were added to LPS during murine or human DC maturation they increased the production of the Th1-inducing cytokine IL-12p70 [34], [35]. Interestingly, IL-4 in equine DC generated from peripheral blood has been shown to mature DC by its own [36]. All of these data indicate IL-4 produced early during an infection by innate immune cells may prime immature DC to switch to immunity or enhance maturation induced by weak stimuli. When IL-4 was added to the human lowGM-DC cultures we obtained mixed results. For some donors IL-4 did not influence the maturation-resistant phenotype, while other cultures lost their maturation-resistance (not shown). Thus, it remains to be determined how the influence of IL-4 (and IL-13) can be counteracted before the clinical use of our lowGM-DC.

Previous reports indicated that repetitive *in vitro* stimulations of human allogeneic T cells with immature Mo-DC resulted in the generation of T regs [37]. Here we report that immature DC generated from BM induced T cell anergy but not T regs. The discrepancy may be explained by different cellular sources of the DC precursors leading to different immature DC phenotypes. In fact, immature Mo-DC express higher levels of HLA-DR and costimulatory molecules, CD80 and CD86, (despite CD83 being negative) as compared to human tissue-resident DC analyzed *ex vivo* from liver [38], epidermis [38], or spleen [39], [40]. In this respect those tissue-derived DC resemble our immature DC generated from BM (both lowGM and highGM/4). T cell anergy induction has been previously shown by several groups to be mediated by immature DC, while T regs require CD28 costimulation to be fully functional. Thus, the CD80/CD86^low^ immature BM-DC may induce anergy, while the CD80/CD86^int^ immature Mo-DC may induce T regs. On the other hand, the induction of T regs is known to depend on the presence or DC production of TGF- β, retinoic acid, or IL-10. In addition immature splenic DC to induce T regs [41]. In this view there may also exist differences between DC derived from different precursors.

Anergy induction in naïve allogeneic T cells by both lowGM-DC and highGM/4-DC required two rounds of stimulation by immature DC. It has been shown effector T cells and T cell clones can be rendered anergic by a single TCR signal in the absence of costimulation, but that *in vivo* suboptimal costimulation via CD80/CD86 may be required to anergize naïve T cells through CTLA-4 [11]. On the other hand *in vivo* examples indicate anergy induction may be induced in the presence of co-stimulation independently of CTLA-4 by blocking the cell cycle [42], [43]. Also inversely, we could show that in the absence of the two major cell cycle inhibitors, p27Kip1 and p21Cip1, T cell anergy could still be induced in mice [44]. Delineated from these data two distinct pathways of T cell anergy induction have been proposed: one via cell cycle inhibition and the other via CTLA-4 [45].

Recent data indicate that CTLA-4 upregulation is mediated via the TCR as shown using CD3 antibodies [46]. Thus, the CD80/CD86 expression by immature or mature DC may not contribute to CTLA-4 upregulation. In a second stimulation immature DC expressing low levels of CD80/CD86 may trigger preferentially CTLA-4 on primed T cells, not CD28, due to a higher affinity of CTLA-4 to its ligands and limited availablity of CD80/CD86 molecules at the surface of immature DC. Although further analysis will be required to show a causal relationship between the CTLA-4 upregulation and the requirement for a second hit by immature DC, our data support a model depicting how T cell anergy can be induced by two stimulations with immature DC, providing a basis for the potential physiological mechanism of T cell anergy induction via CTLA-4 also *in vivo*.

Our data indicate that a second stimulation with mature DC after a first stimulation with immature DC will not lead to T cell anergy. A higher level of CD80/CD86 expression on the surface of mature DC as compared to immature DC, may trigger CD28, as well as, trigger CTLA-4. When both ligands are encountered by T cells, anergy induction may then be prevented by the dominant CD28 signal. Indeed, early *in vitro* experiments investigating the T cell priming by CD3/CD28/CTLA-4 antibody combinations indicated CD28 can outcompete negative CTLA-4 signals depending on their ratio [47]. Alternatively other costimulatory molecules or cytokines expressed by mature DC could interfere with the negative CTLA-4 signal [48] or via control of the spatial and temporal expression kinetics of CD28/CTLA-4 on T cells and CD80/CD86 on DC [49].

In the reverse setting, when mature DC were applied as the first round of stimulation, complete T cell activation may have been initiated involving many other types of costimulatory molecules and cytokines. When immature DC were applied as the second round, they were unable to revert the T cell activation program back into an anergic state.

In conclusion, we provide a protocol that allows the generation of immature and maturation-resistant DC according to GMP guidelines to induce allogeneic transplantation tolerance. Furthermore, by using DC for anergy induction, which represent more physiological conditions as compared with antibody studies, our data allow us to speculate how immature DC could induce T cell anergy by two subsequent stimulations involving CTLA-4 signalling.

## Materials and Methods

### Ethics Statement

BM samples were received from patients with various hematological disorders who were in clinical remission following appropriate therapy and who were seen for routine follow-up that included BM analysis. Informed written consent of all the patients to this study was obtained before BM sample acquisition. BM sampling and the experimental work occurred after approval from the Ethical Committee of the Medical Faculty at the University of Erlangen (approval no. 2856).

### Generation of human BM-DC

Heparinized BM samples were diluted 1∶4 in PBS and layered on a density gradient (Lymphoprep®, Nycomed Pharma AS) and centrifuged (486×g/30 min/20°C). Interphase cells were collected and plated at a density of 5×10^6^ cells/10 cm petri dish (Falcon #1029) in 10 ml R10 medium consisting of RPMI 1640 (Bio-Whittaker (Cambrex, Vervier, Belgium) with heat inactivated 1% AB plasma (kindly provided from the Department of Transfusion Medicine, University of Erlangen), 100 U/ml penicillin, 100 µg/ml streptomycin (PAA) and L-Glutamin (PAA). Generation of human BM-DC followed then largely the protocol established for murine lowGM cultures, described before [23]. Briefly, 4–5.5×10^6^ BM cells per 10 cm petri dish (Falcon, #3003) were seeded and cultured under highGM conditions received 800 U/ml rhGM-CSF plus 250 U/ml rhIL-4 (both Cell Genix, Freiburg, Germany). LowGM conditions contained 5 U/ml rhGM-CSF. At each days 4 and 6 additional 3 ml R10 were added containing the the same low or high doeses of GM-CSF±IL-4. Cells were used for functional assays at day 7. For DC maturation LPS (100 ng/ml, SIGMA), Poly I∶C (50 µg/ml, Pharmacia), CD40L (300 ng/ml) or maturation cocktail, consisting of 10 ng/ml TNF, 13.2 ng/ml IL-1β, 1000 U/ml IL-6, 1 µg/ml PGE_2_ (Cell Genix, Freiburg, Germany) was added over night.

The DC that were used in this study were generated from BM cells of routine follow-up patients with various hematological disorders. The cellular yields and the functional quality of the DC was comparable in all the experiments that were performed, indicating that no disease-specific factors had influenced the BM quality and thereby modulate DC function. Similar data regarding the maturation-resistant DC phenotype by using the protocol for lowGM-DC generation were also obtained from cord blood CD34^+^ cells. These findings further support that the diseases did influence the LowGM-DC generation.

### Mature DC and T cell sources

As a control for the less established BM-DC cultures, Mo-DC were generated as stimulators in the allogeneic T cell assays. For allogeneic T cell stimulation the non-adherent fraction (NAF) of cells from peripheral blood mononuclear cells (PBMC) from healthy donors were used. Peripheral blood samples were treated as the BM cells above to obtain PBMC in the interphase. These cells were then plated at a density of 2×10^6^ per 10 cm petri dish (Falcon #3003) for 1–2 h to obtain adherent monocytes cells for DC generation and the NAF which was used as a source of T cells. The well standardized Mo-DC culture was performed as described in detail before [50].

### Flow cytometry

DC or T cells were harvested and stained for 30 min on ice, each step with the following antibodies: HLA-DR, CD40, CD83, CD86, all conjugated with FITC and CD3-PE (Becton Dickinson). In the experiments where Foxp3 was determined, cells were labelled with carboxyfluorescein-succinimidylester (CFSE, Molecular Probes, 5 µM, 15 min at room temparature) before the stimulation by DC and stained intracellularly with Foxp3-APC conjugate after 4 days of culture according to the manufacturer's descriptions (e-bioscience). Samples were analysed using a FACScan or FACS Canto II (Becton Dickinson). To measure cytokine production the supernatants of the T cell cultures were visualized by cytokine bead array (CBA by BD) according to the manufacturer's recommendations and analysed by a FACScan.

### Allogeneic T cell stimulation

As a T cell source the NAF was used and the cells were cultured for primary stimulations in 24-well plates at 1×10^6^ cells/well together with the indicated maturation stage of allogeneic DC at 1×10^5^/well for 5 days. Aliquots of the respective maturation stage of DC from this donor were stored frozen for subsequent restimulations with the same DC allotype and maturation stage. T cell restimulation by DC occurred by adding 1×10^5^ DC/well. After one or two round of stimulation by DC another the T cell cultures were restimulated as triplicate cultures in flat bottom 96-well plates at 3×10^5^ T cells/well (i.e. NAF) and 3×10^4^ DC/well (initial allotype or third party, as indicated) or with 1000 U/ml rhIL-2 or precoated anti-CD3 alone or precoated anti-CD3 plus soluble anti-CD28 (both 10 µg/ml, Becton Dickinson). After 3 days [^3^H]-thymidine (Amersham) was added overnight and proliferation measured with a 96-well harvester (TOMTEC) and the filters counted in a 1450 Microbeta Counter Wallac-Trilux. Results were acquired with a Microbeta Windows Workstation program and analyzed in Excel.
